# Geographic access to emergency pediatric dental care during COVID-19: a population-based study from Israel

**DOI:** 10.1186/s13584-026-00745-4

**Published:** 2026-01-12

**Authors:** Dana Atia Joachim, Ephraim Shapiro, Thabet Asbi, Doron Haim, Michael V. Joachim

**Affiliations:** 1 Department of Health Systems Management, Ariel University, Ariel, Israel; 2 Maccabi-Dent Research Department, Tel-Aviv, Israel; 3 Department of Periodontology and Implant Dentistry, Rambam Health Care Campus, Haifa, Israel; 4 Department of Periodontology, Dental Research Division, Guarulhos University, Guarulhos, Brazil; 5 Unit of Oral and Maxillofacial Surgery, Shamir (Assaf ha-Rofeh) Medical Center, 730001 Tzrifin, Israel

**Keywords:** Dental care, Healthcare accessibility, Geographic information systems, Pediatrics, COVID-19, Health services research, Emergency services

## Abstract

**Background:**

The COVID-19 pandemic created unprecedented challenges for healthcare systems, particularly affecting specialized services like pediatric dental care. This study examined geographic disparities in emergency dental service access for children during the first COVID-19 lockdown in Israel.

**Methods:**

We analyzed data from a major healthcare provider serving 25% of Israel’s population, comparing 6,024 emergency dental visits of children under 12 across three time periods: pre-pandemic (March-May 2019), first lockdown (March-May 2020), and post-lockdown (March-May 2021). Using spatial analysis methods, we evaluated the distribution and accessibility of emergency dental services across four main geographic regions through metrics including Load Ratio (LR), Geographic Availability Index (GAI), and Rate of Change in Service Utilization (ROCSU).

**Results:**

During the lockdown, emergency visits decreased by 40.2% compared to the pre-pandemic period, with significant regional variations ranging from 31.2% in the Northern region to 44.8% in the Central region. The mean age of children seeking emergency care during lockdown (6.2 years) was significantly lower than in both pre-pandemic (7.1 years) and post-lockdown periods (6.8 years). Analysis revealed substantial regional disparities in service burden, with the highest Load Ratio in the Central region (1.86) and lowest in the Northern region (1.24), despite the Central region having the highest Geographic Availability Index (2.46). The Jerusalem area had the highest proportion of invasive treatments (40.4%) and swelling/abscess cases (22.4%). Ultra-Orthodox neighborhoods demonstrated distinct utilization patterns, with a lower decrease in emergency visits (29.8–36.8%) compared to the national average (40.2%).

**Conclusions:**

This study identified significant geographic inequities in emergency dental care access during crisis periods, with a paradoxical relationship between service availability and utilization across regions. Our analysis suggests that emergency dental service planning should incorporate strategic facility placement to minimize travel barriers, consideration of population-specific utilization patterns, and balanced resource allocation that maintains proportional service capacity across diverse geographic contexts. These findings provide important insights for health system preparedness to ensure equitable access to essential pediatric dental services during future crises.

## Introduction

The COVID-19 pandemic created unprecedented challenges for healthcare systems worldwide, particularly affecting specialized services like pediatric dental care. In Israel, the first case was identified on February 21, 2020, with cumulative cases reaching approximately 4.07 million and a relatively low mortality rate of 0.2% [1, 2]. The Israeli government responded with a series of lockdowns, with the first lockdown imposed on March 14, 2020, involving severe restrictions on movement and public activities, including limitations on healthcare services [3, 4]. The initial lockdown period was followed by additional lockdowns later in 2020 in response to new waves of infection [5, 6].

The dental care sector was significantly impacted due to the inherent transmission risks associated with dental procedures. Dental treatment inherently requires close physical proximity between practitioners and patients, and many procedures generate aerosols that can significantly increase transmission risks [7, 8]. Following specific Ministry of Health guidelines issued on March 17, 2020, dentists were instructed to postpone all non-emergency treatments, limiting dental care to emergency cases only [9]. According to these guidelines, dental clinics were permitted to operate in emergency mode only [10].

Maccabi Healthcare Services, serving approximately 25% of Israel’s population, operates its dental services through Maccabident, which normally runs 53 dental clinics with about 400 dental chairs and over 1,100 dentists. However, during the first lockdown, services were drastically reduced to just 84 dentists across 29 branches, providing emergency care. These emergency dental services addressed both adult and pediatric patients, though no data is available on the specific proportion of resources allocated to pediatric versus adult care during this period. This reduction in service availability created significant challenges for accessing dental care, particularly for vulnerable populations such as children [10, 11].

Children are a population that is particularly vulnerable in dental emergencies, with distinct characteristics that differentiate them from adult patients. Dental emergencies in children present unique challenges due to anatomical differences (thinner enamel and wider root canals in primary teeth), limited ability to communicate pain levels, developing immune systems that may lead to faster infection progression, and potential impacts on normal oral development when treatment is delayed [12]. Research shows that dental pain in children often leads to immediate care-seeking behavior, as young patients have limited ability to cope with pain, and parents tend to respond more urgently to their children’s complaints [13]. Studies conducted during the pandemic revealed that while overall dental visits decreased, the proportion of severe cases and emergency interventions increased significantly, particularly among younger children [14]. In Israel specifically, the risk of hospitalization due to dental infections in children increased 3.28-fold during the pandemic period compared to pre-pandemic levels [15].

Beyond clinical considerations, geographic accessibility emerged as a critical factor affecting emergency dental care during the pandemic. A research study examining the effect of COVID-19 on pediatric dental care in Israel found that dental emergency services were distributed unevenly, with significant disparities between central urban areas and peripheral regions [16]. During the first lockdown, transportation limitations and varying travel times to available clinics created disparate access burdens especially for families living in peripheral areas.

These spatial barriers particularly affected vulnerable populations like children, whose caregivers faced additional challenges navigating limited transportation options and greater distances to functioning emergency facilities [17]. The distribution of service reductions often followed administrative rather than needs-based patterns, potentially amplifying pre-existing geographic disparities in healthcare access [18].

The Ultra-Orthodox (Haredi) Jewish population presents a unique case study for understanding place-based healthcare utilization patterns during emergencies. This community is characterized by distinctive residential clustering in specific neighborhoods and cities such as Bnei Brak and Jerusalem, and the cities of Modi’in Illit and Beitar Illit. Their geographic concentration is accompanied by unique mobility patterns, including lower rates of private vehicle ownership and greater reliance on public transportation, which creates particular vulnerabilities during lockdown periods when public transit is limited. Previous research has identified differential healthcare utilization patterns among Ultra-Orthodox communities, influenced by both cultural factors and geographic accessibility [19]. These communities often demonstrate both underutilization of preventive dental services and higher emergency utilization rates compared to the general population. The intersection of place-based access barriers with the demographic characteristics of this community—including larger family sizes and higher proportions of children—creates a distinctive pattern of emergency dental care needs that warrants specific investigation [14].

The unique circumstances of the first lockdown provide an exceptional opportunity to examine the minimum threshold of emergency dental services required for the pediatric population. Geographic accessibility to these services becomes particularly crucial during crisis periods, as travel restrictions and reduced service availability can create significant barriers to care. This study aims to analyze the spatial distribution and accessibility of emergency dental services for children during the first COVID-19 lockdown in Israel, focusing on identifying minimal resource requirements and geographic disparities in service access. Understanding these patterns is essential for future emergency preparedness and the development of more resilient healthcare systems that can maintain essential services during crises.

## Methods

### Ethics considerations

This study was approved by the Institutional Helsinki Committee of Maccabi Healthcare Services (approval #MHS-0079-24). The committee determined that the research complies with the principles of the Declaration of Helsinki and the Public Health Regulations. As this was a retrospective study based on existing medical records, the committee granted a waiver of informed consent. All data were fully anonymized prior to analysis to preserve patient confidentiality.

### Study design and data source

This retrospective observational study analyzed data from the computerized medical records system of Maccabident, a dental service provider under Maccabi Healthcare Services, which serves approximately 25% of Israel’s population. The study examined three parallel time periods: the first COVID-19 lockdown period (March 25 - May 4, 2020), the equivalent period in the preceding year (March 25 - May 4, 2019), and the equivalent period in the subsequent year (March 25 - May 4, 2021). All data were fully anonymized, with identifying information such as names, identification numbers, and precise addresses removed while preserving relevant variables, including age, gender, and geographic region.

### Study population

The study population included all children under 12 years of age who sought emergency dental treatment at Maccabident clinics during the three periods examined. This age limit was selected because patients over 12 typically present with permanent teeth only, with dental characteristics similar to adults. Inclusion criteria required patients to be under 12 years old at the time of treatment and to have complete documentation in the patient’s computerized medical record.

### Emergency service protocol during lockdown

During the first lockdown period, access to dental care was provided through a dedicated telephone triage system. Information collected included the reason for emergency consultation, the patient’s general health status, and COVID-19 status (exposure or symptoms). A team of dentists assessed each complaint, reviewed the patient’s dental records, and scheduled appointments at the nearest operational dental clinic for qualifying patients. Patients with suspected COVID-19 infection were referred for testing and not treated until a negative result was confirmed.

### Geographic distribution framework

The geographic analysis was conducted using a framework based on established health service regional classification [18]. Dental clinics were categorized into four main geographic regions based on Israel’s major population centers and administrative health districts:

1. Northern region (9 clinics): Serving Haifa, the Krayot, and extending to Nof HaGalil and Zichron Ya’akov.
2. Central region (27 clinics): Serving the greater Tel Aviv metropolitan area from Netanya in the north to Rishon LeZion in the south, and from Modi’in in the east to Herzliya in the west.
3. Jerusalem and surrounding areas (6 clinics): Serving greater Jerusalem, Beit Shemesh, and Beitar Illit.
4. Southern region (8 clinics): Extending from Ashdod in the north to Eilat in the south.

During the first lockdown, approximately half of the clinics in each region remained operational. The selection of which clinics would remain open was based on population distribution and geographic coverage considerations. This resulted in 5 operational clinics in the Northern region, 14 in the Central region, 3 in the Jerusalem area, and 4 in the Southern region.

### Spatial accessibility analysis

To evaluate the efficiency of the geographic distribution of active clinics during the lockdown and to quantify geographic disparities in service access, three established metrics from healthcare accessibility literature were employed:

1. Load Ratio (LR): [20] This metric quantifies the relative burden on clinics that remained operational during the lockdown compared to pre-pandemic operations. It was calculated using the formula:
  $$\:LR=\frac{Vl*Ct}{Vr*Cl}$$
  where Vl represents the number of visits during the lockdown, Vr represents visits during routine periods, Ct is the total number of clinics in the region, and Cl is the number of active clinics during the lockdown. A Load Ratio greater than 1 indicates a higher relative burden on active clinics during the lockdown.
2. Geographic Availability Index (GAI): [17] This metric evaluates the spatial availability of emergency dental services relative to population needs. It was calculated as:
  $$\:GAI=\frac{Cl}{V}*100$$
  where Cl is the number of active clinics during the lockdown, and V is the average daily number of visits in the region during the lockdown. Higher values indicate better availability of services relative to demand.
3. Rate of Change in Service Utilization (ROCSU): [21] This metric assesses changes in healthcare service utilization patterns during the COVID-19 pandemic. It was calculated as:
  $$\:ROCSU=\left(\frac{Vr-Vl}{Vr}\right)\text{*}100\:$$
  where Vr and Vl represent the number of visits in regular periods and during lockdown, respectively. Higher percentages indicate greater reduction in service utilization.

### Ultra-Orthodox population analysis

This study specifically examined the Ultra-Orthodox (Haredi) Jewish subgroup due to our ability to identify this demographic group through geographic location analysis. Ultra-Orthodox patients are identifiable through their concentration in specific neighborhoods and cities, enabling analysis of service utilization patterns without requiring individual-level religious affiliation data, which is not typically recorded in medical records. To examine the impact of the pandemic on dental services use for this specific sub-population of children, clinics serving Haredi populations were identified based on their geographical location in predominantly Haredi cities (Modi’in Illit, Beitar Illit and Elad) and in mixed cities with substantial Haredi populations (Bnei Brak - Rabbi Akiva and Kahanneman clinics; Beit Shemesh - G and Naimi Center clinics; Jerusalem - Agripas and Pisgat Ze’ev clinics). During the lockdown period, service availability in these areas was limited, with only Rabbi Akiva clinic in Bnei Brak, Agripas clinic in Jerusalem, and clinics in Haredi cities remaining operational. While other minority populations in Israel, particularly Arab communities, also have distinct residential patterns and unique healthcare utilization behaviors, methodological limitations prevented their inclusion in the current analysis. Unlike Ultra-Orthodox communities, Maccabident clinics serving Arab communities are typically located in mixed municipalities or Jewish-majority cities with adjacent Arab communities, where population identification would require manual review of individual records based on names or other identifiers, resources for which were not available within the scope of this study.

### Variables and data collection

Demographic data collected included age, sex, and residential area (North, Central, Jerusalem area, or South).

Treatment variables were categorized into two main groups:

**1. Reason for emergency visit:**

• **Pain of pulpal origin**: Acute pain from deep caries, pulpitis, or irreversible pulp inflammation.

• **Soft tissue pain**: Pain from gingivitis, periodontitis, ulcers, or other mucosal lesions.

• **Dental trauma**: Tooth fractures, avulsions, luxations, or other trauma-related emergencies.

• **Swelling/abscess**: Intraoral or facial swelling, abscess formation, or spreading infections.

• **Treatment repair**: Dislodged restorations, broken prostheses, or orthodontic emergencies.

**2. Type of treatment provided:**

• **Medication only**: Prescription of analgesics, antibiotics, or other medications without direct dental intervention.

• **Temporary restoration**: Provisional fillings or temporary coverage of exposed dentin.

• **Permanent restoration**: Definitive restorative procedures including fillings and crowns.

• **Pulp extirpation**: Removal of dental pulp tissue as part of emergency endodontic treatment.

• **Extraction**: Complete removal of a tooth when restoration was not feasible.

• **Drainage**: Incision and drainage procedures for abscesses or other collections of fluid.

These categories were determined by dentists during the initial telephone triage and confirmed during in-person examination. The classification system was based on established guidelines for dental emergency categorization from the American Dental Association and the Israeli Dental Association, adapted for the specific needs of the pediatric population and the emergency conditions of the pandemic.

All information was systematically recorded in the electronic health record system at the time of treatment, ensuring consistent documentation across all clinics and time periods. For analysis purposes, treatments were further classified as “invasive” (extractions and pulp extirpation) or “conservative” (all other treatments) to allow for comparisons between treatment approaches across different regions and time periods.

### Statistical analysis

Statistical analysis was performed using R software (version 4.2.0, R Core Team, 2022, Vienna, Austria), utilizing several specialized packages including stats, dplyr, and tidyr for basic descriptive analyses; car and emmeans for ANOVA and post-hoc comparisons; gmodels for chi-square tests; MASS for multivariate analyses; and ggplot2 for visualization.

A comparative analysis of visit volumes across different periods was conducted using one-way ANOVA with post-hoc Tukey HSD tests. Comparison of demographic characteristics between periods was performed using chi-square tests for independence for categorical variables and an ANOVA test for continuous variables.

Comparisons of spatial accessibility metrics (Load Ratio, Geographic Availability Index, and Rate of Change in Service Utilization) between regions were analyzed using one-way ANOVA with post-hoc Tukey HSD tests to identify significant pairwise differences.

Based on preliminary data, a minimum sample size of 1,200 emergency visits was required to detect a 15% difference in utilization rates between geographic regions with 80% power and α = 0.05. With 6,024 total visits, including 1,393 during the lockdown period, the study had sufficient statistical power to detect even smaller regional differences.

For all statistical tests, p-values less than 0.05 were considered statistically significant. For multiple comparisons, a Bonferroni correction was applied by adjusting the significance threshold to α/n, where n represents the number of comparisons, to control for family-wise error rate.

## Results

### Population characteristics and geographic distribution of emergency visits

This study analyzed 6,024 emergency dental visits of children under 12 years of age across three time periods. During the pre-pandemic period (March-May 2019), 2,331 emergency visits were recorded. The lockdown period (March-May 2020) showed a substantial decrease, with 1,393 emergency visits, representing a 40.2% reduction. In the post-lockdown period (March-May 2021), visit volumes returned to pre-pandemic levels with 2,300 visits.

The reduction in emergency visits during the lockdown varied considerably by geographic region (Table 1). The Central region experienced the most substantial decline (44.8%, from 1,028 to 568 visits), while the Northern region showed the smallest reduction (31.2%, from 393 to 282 visits). Intermediate decreases were observed in the Jerusalem area (42.5%, from 436 to 251 visits) and Southern region (38.4%, from 474 to 292 visits).

**Table 1:** Comparison of visit volumes and decline rates by geographic region

Region	Pre-pandemic visits	Lockdown visits	Post-lockdown visits	Percent decline during lockdown
North	393	282	393	31.2%
Central	1,028	568	997	44.8%
Jerusalem	436	251	442	42.5%
South	474	292	468	38.4%
**Total**	**2**,**331**	**1**,**393**	**2**,**300**	**40.2%**

Bold values indicates total numbers

An analysis of emergency visit patterns by region during lockdown revealed distinct geographic variations in visit reasons (Table 2). The Northern region had the highest proportion of pain of pulpal origin emergency visits (48.4% of all visits), compared to 41.2% in the Central region, 39.6% in the Jerusalem area, and 40.8% in the Southern region (*p* < 0.001). Swelling and abscess-related emergencies were most prevalent in the Jerusalem area (22.4% of visits), as compared to 17.6% in the Central region and 18.2% in the Northern region (*p* = 0.02).

**Table 2:** Regional variations in emergency visit reasons during lockdown period (March-May 2020)

Visit Reason	North (*n* = 282)	Central (*n* = 568)	Jerusalem (*n* = 251)	South (*n* = 292)	*p*-value
Pain-related	136 (48.4%)	234 (41.2%)	99 (39.6%)	119 (40.8%)	< 0.001*
Swelling/abscess	51 (18.2%)	100 (17.6%)	56 (22.4%)	59 (20.1%)	0.02*
Trauma-related	20 (7.1%)	53 (9.4%)	19 (7.6%)	24 (8.2%)	< 0.001*
Soft tissue pain	44 (15.6%)	91 (16.1%)	40 (15.9%)	45 (15.4%)	0.72
Treatment repair	30 (10.7%)	89 (15.7%)	36 (14.5%)	45 (15.5%)	0.04*

*Statistically significant (*p* < 0.05), chi-square test for independence

### Spatial accessibility metrics

**Fig. 1:**
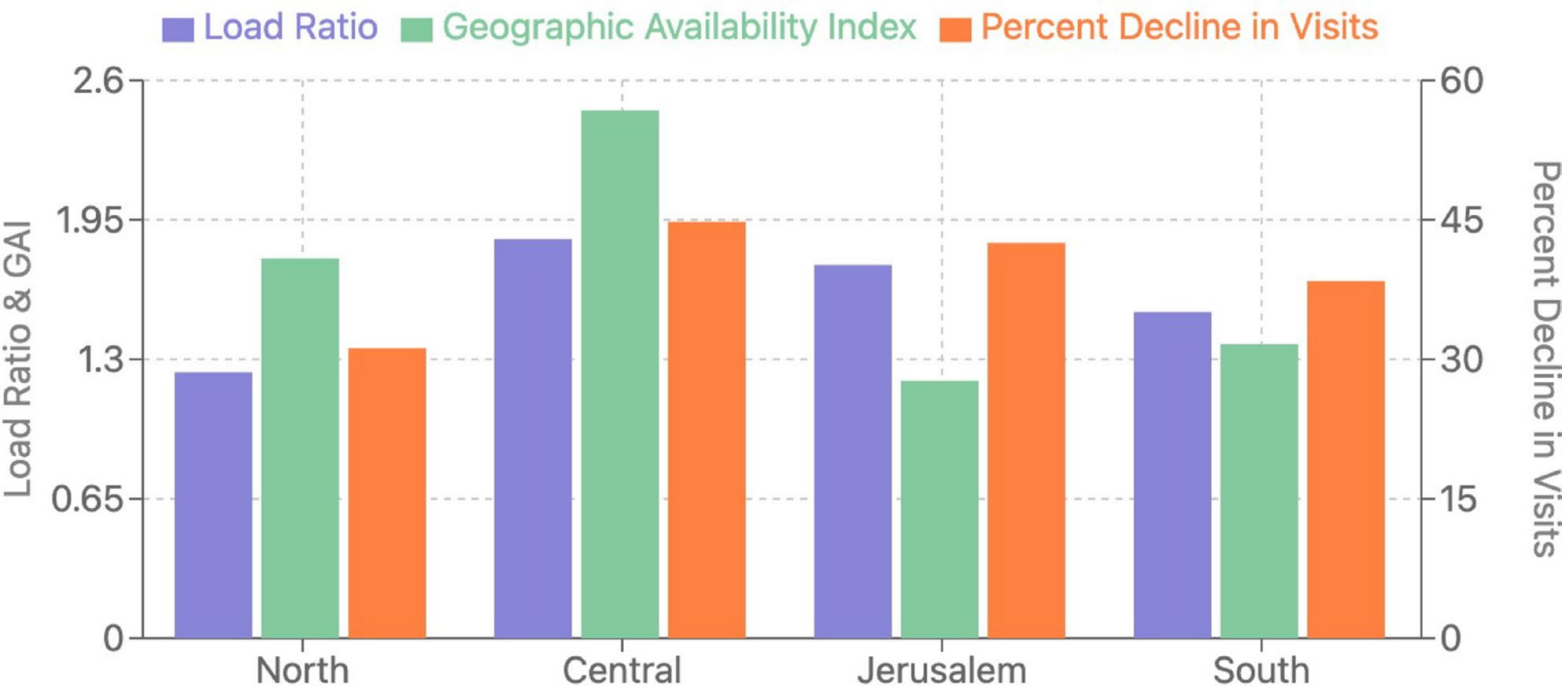
Spatial Accessibility Metrics by Geographic Region During COVID-19 Lockdown. Bar chart comparing Load Ratio (LR), Geographic Availability Index (GAI), and Rate of Change in Service Utilization (ROCSU) across the four geographic regions of Israel (North, Central, Jerusalem, and South)

Analysis of spatial accessibility metrics revealed significant geographic disparities in service access during the lockdown period (Table 3). The Load Ratio (LR) showed substantial differences between regions (F = 18.7, *p* < 0.001), with the Central region experiencing the highest service burden (LR = 1.86), significantly higher than the Northern region (LR = 1.24, *p* < 0.001) and the Southern region (LR = 1.52, *p* = 0.02). The differences between the Central and Jerusalem regions (LR = 1.74) were not statistically significant (*p* = 0.31) (Fig. 1).

The Geographic Availability Index (GAI) also varied significantly across regions (F = 14.3, *p* < 0.001), with post-hoc analysis revealing significant differences between the Central region (GAI = 2.46) and both the Jerusalem area (GAI = 1.20, *p* < 0.001) and Southern region (GAI = 1.37, *p* < 0.01), while the difference between Central and Northern regions (GAI = 1.77) approached but did not reach statistical significance (*p* = 0.06) (Fig. 1).

**Fig. 2:**
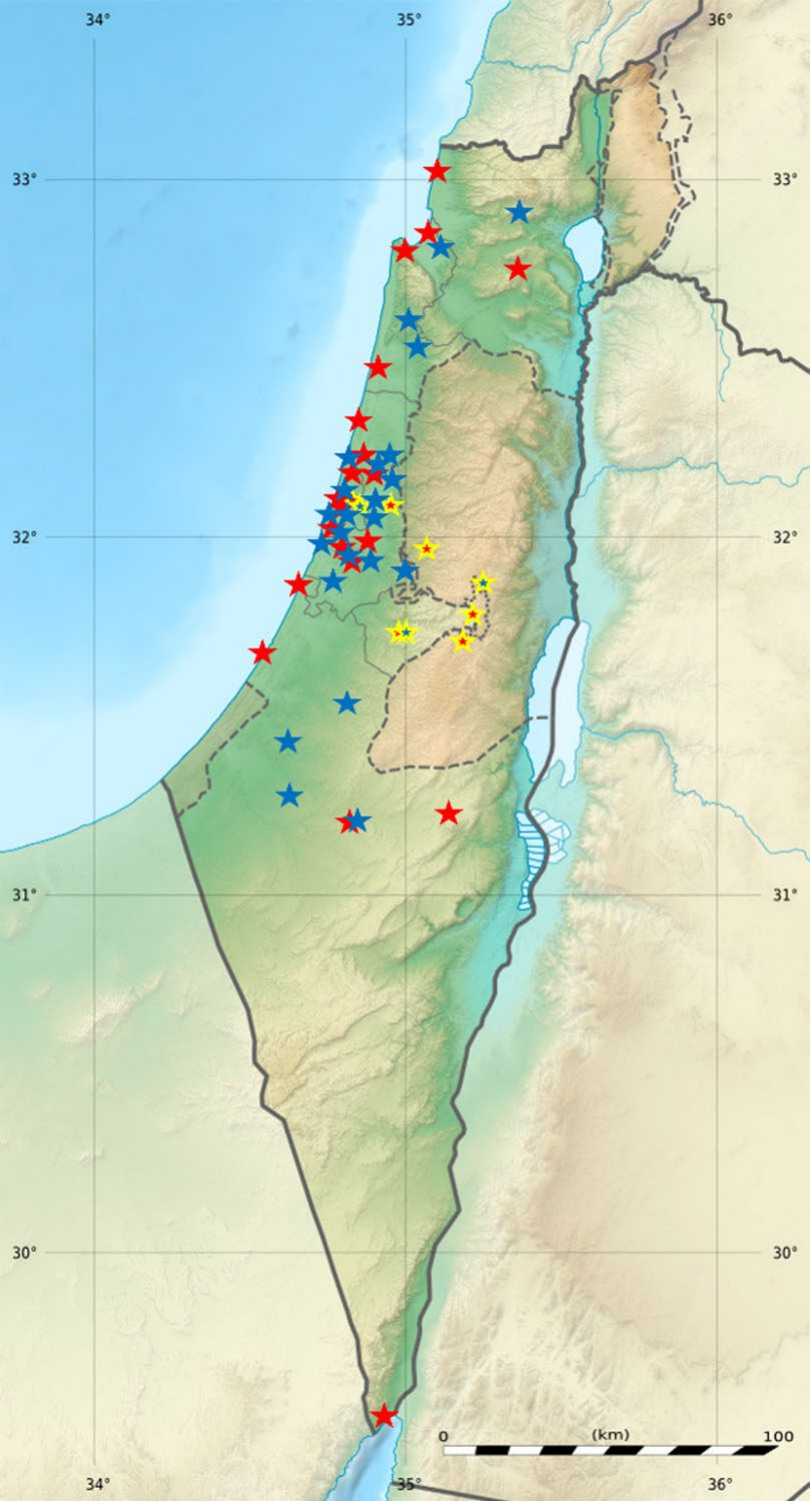
Geographic Distribution of Emergency Dental Clinics Before and During the COVID-19 Lockdown in Israel. Map of Israel showing the distribution of all dental clinics (*n* = 53) before the pandemic and those that remained operational (*n* = 26, marked in red) during the first lockdown period (March-May 2020). Ultra-Orthodox areas highlighted with gold borders. Base relief map adapted from Wikimedia Commons, licensed under CC BY-SA 3.0 (Author: NordNordWest)

The Rate of Change in Service Utilization (ROCSU) analysis showed significant regional variations in emergency visit reductions (F = 16.2, *p* < 0.001). The Central region exhibited the highest decrease (−44.8%), significantly greater than the Northern region (−31.2%, *p* < 0.001) and the Southern region (−38.4%, *p* = 0.02). The Jerusalem area’s decrease (−42.5%) was not significantly different from the Central region (*p* = 0.31) but was significantly higher than the Northern region (*p* < 0.001). These variations in ROCSU values indicate substantial regional differences in emergency service continuity during the lockdown period. The spatial distribution of operational clinics (Fig. 2) illustrates how these regional disparities manifested geographically.

**Table 3:** Spatial accessibility metrics by geographic region during lockdown

Region	Load ratio	Geographic availability index	Rate of change in visits (%)	Active clinics during lockdown	Total clinics
North	1.24	1.77	−31.2%	5	9
Central	1.86	2.46	−44.8%	14	27
Jerusalem	1.74	1.20	−42.5%	3	6
South	1.52	1.37	−38.4%	4	8

### Ultra-Orthodox population service utilization

An analysis of clinics serving predominantly Ultra-Orthodox populations revealed distinct utilization patterns (Table 4). Despite service reductions, clinics in Ultra-Orthodox areas experienced a smaller decrease in emergency visits compared to the national average. In Bnei Brak, visits decreased by 32.4%, significantly less than the national average of 40.2%, despite the closure of the Kahanneman clinic and the operation of only Rabbi Akiva clinic. Similar patterns were observed in Beit Shemesh (36.8% decrease), Modi’in Illit (34.1% decrease), Beitar Illit (33.2% decrease), Elad (35.5% decrease), and at the Agripas clinic in Jerusalem (29.8% decrease).

**Table 4:** Service utilization in Ultra-Orthodox areas during lockdown

City	Active clinics during lockdown	Regular Clinics	Average Daily Visits During Lockdown	Average Daily Visits Pre-Pandemic	Percent Decrease
Bnei Brak	1	2	9.7	14.8	32.4%
Beit Shemesh	1	2	7.7	11.2	36.8%
Modi’in Illit	1	1	7.2	9.6	34.1%
Beitar Illit	1	1	7.4	9.8	33.2%
Elad	1	1	7.0	10.9	35.5%
Jerusalem (Agripas)	1	2	8.8	12.4	29.8%

All Ultra-Orthodox locations are within the Jerusalem and Central regions as defined in our geographic framework. The Ultra-Orthodox cities of Modi’in Illit and Beitar Illit are located in the Jerusalem region, while Bnei Brak and Elad are in the Central region, and Beit Shemesh is on the border between the Jerusalem and Central regions.

Despite the overall decrease in visits, the Rabbi Akiva clinic in Bnei Brak experienced increased service pressure as it became the sole operational clinic in the area, with its Load Ratio (1.52) indicating significant strain on resources. Similarly, the Agripas clinic in Jerusalem faced heightened service demands with a Load Ratio of 1.41. The Elad clinic maintained operations throughout the lockdown period, showing a Load Ratio (1.41) comparable to other Ultra-Orthodox locations. When examining overall service metrics, Ultra-Orthodox areas maintained better service continuity with relatively lower Load Ratios (ranging from 1.33 to 1.52) compared to the overall regional averages, particularly the Central region (1.86).

### Treatment patterns across regions

Examination of treatment patterns revealed significant regional differences in the types of emergency interventions provided during the lockdown period. The Jerusalem region had the highest proportion of invasive treatments (extraction and pulp therapy) at 40.4% of all treatments, compared to 32.4% in the Central region, 29.8% in the Northern region, and 34.2% in the Southern region (*p* < 0.001).

Across all regions, there was a shift from conservative treatments to more invasive procedures during the lockdown period. The proportion of medication-only treatments decreased from 44.6% pre-pandemic to 22.4% during lockdown (*p* < 0.001), while the proportion of extractions increased from 18.4% to 24.8% (*p* < 0.001). This shift likely reflects the higher threshold for seeking care during the lockdown period, with families delaying visits until conditions became more severe and required more invasive interventions.

## Discussion

This study provides critical insights into geographic disparities in emergency dental service access for children during a period of extreme healthcare system stress. By examining spatial distribution patterns and accessibility metrics during the COVID-19 lockdown in Israel, we identified significant geographic inequities that impacted healthcare delivery and utilization. Our findings contribute to an expanding body of literature on crisis-induced healthcare disparities and have important implications for emergency preparedness planning.

### Geographic disparities in service reduction and utilization

The substantial variation in service utilization decline between geographic regions (ranging from 31.2% in the Northern region to 44.8% in the Central region) reveals how crisis situations can exacerbate existing regional disparities. While our initial expectation might be that more densely populated central areas would maintain better service continuity, our findings demonstrate a paradoxical relationship between theoretical service availability and actual utilization. Despite the Central region having the highest Geographic Availability Index (GAI = 2.46), it experienced the most significant reduction in service utilization (ROCSU=−44.8%).

This inverse relationship between service availability and utilization contradicts conventional healthcare accessibility theories that suggest greater availability correlates with increased utilization [17]. Our findings align instead with research showing that during crises, factors beyond pure geographic proximity significantly influence healthcare access [18]. These factors include transportation limitations, communication effectiveness regarding available services, and socioeconomic barriers—all of which may be amplified during emergency situations.

The paradoxical finding that the Central region experienced the greatest reduction in service utilization despite having the highest Geographic Availability Index can be explained by several factors. First, the greater urbanization and population density in central Israel likely intensified COVID-19 transmission concerns [1], with evidence suggesting that densely populated areas experienced higher infection rates during the first wave [6]. Second, the Central region has a higher concentration of private dental practices, potentially providing alternative care options not captured in our dataset [22]. Third, transportation disruptions during the lockdown disproportionately affected urban areas that are more reliant on public transportation [18], creating additional access barriers despite geographic proximity to services. Finally, the stricter enforcement of lockdown measures in the Central region compared to peripheral areas may have contributed to the greater reduction in dental visits.

The regional variations observed in our study are consistent with findings from research in other countries. Studies in Iran reported that the impact of COVID-19 on dental service utilization was more severe in public sector facilities, which predominantly serve lower socioeconomic populations [23]. Similarly, research in the United States found persistent gaps in dental care utilization between privately and publicly insured patients during the pandemic [21]. These parallels suggest that the geographic disparities we identified likely intersect with socioeconomic factors, creating compound vulnerabilities for certain populations.

### Service burden and resource distribution

The Load Ratio analysis revealed significant differences in service burden across regions, with the Central region experiencing the highest pressure on remaining facilities (LR = 1.86) compared to the Northern region (LR = 1.24). This disparity indicates suboptimal resource distribution during the emergency period. The higher Load Ratio in the Central region suggests that despite having more clinics in absolute numbers, the proportional reduction in services created disproportionate strain on the remaining facilities.

Previous studies on healthcare resource allocation during disasters have emphasized the importance of strategic facility distribution based on population density and travel time considerations [20]. Our findings highlight the challenges in achieving balanced resource distribution during rapid service contraction. The Jerusalem area, with the lowest Geographic Availability Index (GAI = 1.20) and a high Load Ratio (LR = 1.74), exemplifies how inadequate emergency service planning can create critical access barriers, which is particularly concerning given this region’s high proportion of severe cases requiring immediate intervention (22.4% swelling/abscess cases).

Such geographic disparities in service burden have been documented in other healthcare contexts during the pandemic. Research in Italy showed that emergency dental admissions in northern regions faced significantly higher burdens than in central and southern regions during lockdown periods [24]. Similarly, studies in Germany found greater impact on larger dental practices compared to smaller ones, suggesting that size and organizational structure influence resilience during crises [22].

### Population diversity and service utilization patterns

#### Ultra-Orthodox communities

Our analysis of Ultra-Orthodox communities reveals important patterns in emergency dental service utilization. The relatively lower decline in service utilization in Ultra-Orthodox areas (29.8–36.8%) compared to the national average (40.2%) suggests distinct healthcare-seeking behaviors. This finding warrants a multifaceted interpretation beyond cultural factors alone.

Several interrelated factors may explain this pattern. First, demographic composition plays a critical role—Ultra-Orthodox communities have larger family sizes with higher proportions of children [25], increasing the baseline need for pediatric dental services. Second, pre-existing utilization patterns are relevant; research has documented lower utilization of preventive dental services in this population [19], potentially leading to more acute care needs that cannot be postponed even during crises. Third, geographic concentration in specific neighborhoods creates unique transportation dynamics—with greater reliance on walking and proximity to services within defined community boundaries [26].

The combination of lower routine care utilization with a smaller decrease in emergency visits suggests that when dental problems reach emergency status in these communities, care-seeking becomes more imperative despite access barriers [13]. This pattern aligns with research showing that the urgency threshold for seeking emergency care varies significantly across population groups [14].

#### Other population groups

While our methodology enabled specific analysis of Ultra-Orthodox communities, it’s important to acknowledge that other minority populations in Israel, particularly Arab communities, may exhibit distinct patterns that merit similar investigation. Arab populations in Israel, comprising approximately 21% of the population, often face unique healthcare access barriers including geographic peripherality, language barriers, and socioeconomic factors [16].

The geographic distribution of Arab communities in Israel—concentrated in the Northern and Southern regions—coincides with our finding of lower service reduction in the Northern region (31.2%). This pattern may partially reflect the healthcare utilization behaviors of this population group, though our methodology does not allow for definitive conclusions. Future research specifically addressing the emergency dental care patterns of Arab communities is needed, particularly given evidence from other healthcare domains suggesting disparities in service access during crisis periods [27].

Similarly, other demographic groups including immigrants from various countries, vulnerable socioeconomic populations, and religious minorities likely experienced differential impacts during the lockdown period. The underlying reasons for regional differences in our findings may partially reflect the diverse composition of Israel’s population across geographic regions.

#### Clinical implications of geographic disparities

Beyond access metrics, our findings revealed concerning clinical patterns that varied by region. The Jerusalem region had the highest proportion of invasive treatments at 40.4% of all emergency interventions, compared to 32.4% in the Central region and 29.8% in the Northern region. This regional variation in treatment intensity suggests that access barriers may have resulted in delayed care-seeking until conditions worsened significantly, necessitating more invasive interventions.

The shift from conservative to invasive procedures observed across all regions during the lockdown (with extractions increasing from 18.4% to 24.8%) aligns with findings from Poland showing a dramatic increase in abscess drainage procedures during lockdown periods [15]. This pattern suggests that dental conditions were allowed to progress to more severe stages before treatment was sought, likely due to a combination of service access barriers and heightened concern about COVID-19 exposure.

The clinical implications of these findings are particularly concerning for pediatric patients. Delayed treatment of dental infections in children has been associated with increased risk of hospitalization, with research showing the risk increased 3.28-fold during the pandemic period [15]. The long-term consequences of increased extractions and reduced conservative treatments include potential impacts on oral development, speech, and long-term dental health [12].

### Implications for policy and emergency preparedness

Our findings have significant implications for emergency preparedness planning in pediatric dental services. The spatial analysis demonstrates that purely population-based or distance-based service reduction approaches may not adequately address actual emergency needs during crises. Instead, resource allocation should consider regional differences in utilization patterns, demographic characteristics, and the spatial distribution of vulnerable populations.

Our data revealed important relationships between regional service distribution and utilization patterns. The Northern region, which maintained the most balanced ratio of operational clinics to emergency demand (LR = 1.24), experienced the lowest reduction in service utilization (−31.2%). This suggests that maintaining appropriate service capacity relative to population needs is critical during emergencies. The high Load Ratio in the Central region (LR = 1.86) indicates that despite having more clinics in absolute numbers, the proportional reduction created excessive strain on remaining facilities.

### Evidence-Based emergency preparedness guidelines

Our spatial analysis provides specific benchmarks for emergency pediatric dental service planning:

#### Service distribution thresholds

The Northern region, which maintained 5 of 9 clinics operational (56% capacity) and achieved the lowest Load Ratio (1.24), experienced the smallest service reduction (31.2%). This suggests that maintaining over 50% of normal clinic capacity helps prevent excessive service strain. In contrast, regions with Load Ratios exceeding 1.8, as observed in the Central region (LR = 1.86), experienced greater access challenges.

#### Geographic coverage standards

Based on our regional analysis, emergency services should prevent service reductions exceeding 45%, as observed in the Central region (44.8% reduction), which correlated with the highest service burden. Strategic distribution of emergency facilities should minimize travel barriers, particularly in peripheral regions [18, 28].

#### Population-Specific resource allocation

Ultra-Orthodox areas demonstrated more resilient utilization patterns, with smaller service reductions (ranging from 29.8% in Jerusalem’s Agripas clinic to 36.8% in Beit Shemesh) compared to the national average (40.2%). This indicates that demographic composition and community characteristics should inform emergency resource allocation strategies.

#### Quality monitoring

Our findings suggest that monitoring treatment patterns can indicate access adequacy. The shift toward more invasive treatments during lockdown (extractions increased from 18.4% to 24.8% nationally) likely reflects delayed care-seeking. Regional variations in treatment complexity should be tracked as indicators of service accessibility.

Emergency dental service planning should therefore incorporate four key principles: (1) regular mapping of pediatric population distribution to identify high-need areas, (2) strategic facility distribution that maintains Load Ratios below 1.8 to prevent excessive service burden, (3) demographic-informed resource allocation that accounts for community-specific utilization patterns, and (4) real-time monitoring of treatment complexity patterns as early indicators of access barriers.

This approach aligns with established healthcare planning frameworks that emphasize the importance of geographic accessibility in emergency service provision [18, 28]. The distinct utilization patterns observed across different population groups underscore the importance of culturally informed approaches to emergency service planning. This principle extends beyond Ultra-Orthodox populations to all demographic groups in Israel’s diverse society. Targeted communication strategies and service distribution that account for demographic, linguistic, and cultural factors would likely improve emergency service utilization equity. Previous research has demonstrated that culturally adapted healthcare delivery models lead to better outcomes during crisis periods for diverse populations [29].

### Study limitations and future research

Several limitations should be considered when interpreting our findings. First, our analysis is based on data from a single healthcare provider, albeit one serving a significant portion (25%) of Israel’s population. While Maccabi Healthcare Services serves diverse populations across Israel, patterns may differ in other healthcare systems. Second, our accessibility metrics focus on geographic and spatial factors and do not fully capture other important dimensions of healthcare access, such as affordability, accommodation, and acceptability.

An additional limitation is the lack of data on the specific capacity allocated exclusively to pediatric emergency dental care during the lockdown period. While our analysis focuses on pediatric emergency visits, the dental clinics that remained operational served both pediatric and adult populations. The absence of data on dedicated pediatric resources (chairs, practitioners, or time allocations) limits our ability to fully assess the adequacy of pediatric-specific emergency dental care capacity during the crisis. This limitation could potentially affect our interpretation of regional disparities, as the observed utilization patterns reflect competition for shared resources between pediatric and adult emergency cases. However, our findings remain valid as they document actual utilization patterns and real access barriers experienced by families seeking pediatric emergency dental care. Future studies would benefit from differentiated capacity data to better evaluate pediatric-specific emergency response planning.

The methodology for identifying population subgroups, particularly the Ultra-Orthodox community, presents a limitation. Our geographic approach to identifying Ultra-Orthodox communities based on clinic location introduces potential misclassification, especially in mixed cities where both Ultra-Orthodox and non-Ultra-Orthodox patients might use the same facilities. Additionally, our methodology did not allow for the examination of other minority groups, particularly Arab populations, who may have experienced unique access challenges during the lockdown period.

Future research should address these limitations through several approaches. First, comparative studies across multiple healthcare providers would provide a more comprehensive picture of emergency dental service utilization patterns. Second, mixed-methods research incorporating qualitative data on patient experiences would enrich our understanding of access barriers beyond geographic factors. Finally, longitudinal studies examining the long-term oral health consequences of emergency care disparities during crisis periods are needed to fully assess the impact of the patterns identified in this study.

Additionally, extending this geographic analysis approach to other minority groups in Israel—including Arab communities, Russian immigrants, Ethiopian Jews, and other culturally distinctive populations—would provide valuable insights into how different demographic groups interact with emergency healthcare systems during crises. Each of these populations potentially has unique spatial distribution patterns, cultural factors affecting healthcare utilization, and specific barriers to accessing emergency services that merit targeted investigation.

## Conclusion

This study revealed significant geographic disparities in children’s emergency dental care access during the COVID-19 lockdown in Israel, with regional variations in service reduction ranging from 31.2% in the Northern region to 44.8% in the Central region. The Load Ratio analysis demonstrated substantial differences in service burden across regions (Northern: 1.24, Central: 1.86), with the Northern region’s more balanced distribution correlating with better service continuity. Regional variations in treatment patterns—with the Jerusalem region showing the highest proportion of invasive treatments (40.4%)—further underscore the clinical implications of these disparities. Our findings emphasize the need for emergency preparedness strategies that account for both geographic accessibility and population-specific utilization patterns, rather than relying solely on population density or administrative boundaries. Future emergency dental service planning should incorporate regular mapping of pediatric population distribution, strategic facility placement to minimize travel barriers, consideration of regional utilization patterns, and balanced resource allocation that maintains proportional service capacity across diverse geographic and demographic contexts.

## Data Availability

The datasets used and/or analyzed during the current study are available from the corresponding author on reasonable request, subject to approval from Maccabi Healthcare Services to ensure patient privacy protections.

## Abbreviations

LR: Load Ratio
GAI: Geographic Availability Index
ROCSU: Rate of Change in Service Utilization
COVID-19: Coronavirus Disease 2019
